# Utilizing the *All of Us* Dataset to Assess the Socioeconomic and Health Impacts of COVID-19 on Hispanics in the United States

**DOI:** 10.3390/ijerph23070859

**Published:** 2026-06-30

**Authors:** William O. Agyapong, Amy Wagler, Bryan J. Castro, Kyle Melin

**Affiliations:** 1 Department of Mathematical Sciences, The University of Texas at El Paso, El Paso, TX 79968, USA; wagyapong@laureateinstitute.org; 2 Laureate Institute for Brain Research, Tulsa, OK 74136, USA; 3 Research Computing and Data Science, New Mexico State University, Las Cruces, NM 88003, USA; awagler@nmsu.edu; 4 National Alliance for Hispanic Health, Washington, DC 20036, USA; bcastro@healthyamericas.org; 5 School of Pharmacy, Medical Sciences Campus, University of Puerto Rico, San Juan, PR 00931, USA

**Keywords:** healthcare disparities, social determinants of health, Hispanic or Latino, health equity, COVID-19

## Abstract

**Highlights:**

**Public health relevance—How does this work relate to a public health issue?**

- Hispanic individuals in the United States disproportionately faced significant negative health and economic outcomes during the COVID-19 pandemic.
- The current study assessed several health and economic effects on Hispanic individuals early in the pandemic, accounting for various social determinants of health.

**Public health significance—Why is this work of significance to public health?**

- Findings support prior evidence that Hispanic individuals in the United States faced higher rates of health and economic impact during the COVID-19 pandemic.
- Iterative proportional fitting (raking) was used to address representativeness and this NIH *All of Us* Driver Project provides a model for addressing limitations in a large, non-probability biomedical dataset.

**Public health implications—What are the key implications or messages for practitioners, policy makers and/or researchers in public health?**

- Public health strategies to reach Hispanic individuals during outbreaks and pandemics must incorporate culturally and linguistically appropriate messaging and channels, to mitigate health and economic effects.
- Given how social determinants of health can intensify effects from the pandemic, public health efforts should focus on individuals who have fewer resources for prevention and treatment.

**Abstract:**

Background. Hispanic populations in the United States experienced disproportionate health and economic impacts during the COVID-19 pandemic. This study assessed relationships between social determinants of health (SDOH) and COVID-19-related health and economic outcomes among Hispanic and non-Hispanic participants in the *All of Us* Research Program. Methods. Descriptive analyses and logistic regression models explored associations between all variables. Iterative proportional fitting (raking) was used to align survey samples with known population margins. Results. Hispanics reported worse outcomes across all COVID-19-related variables: lower vaccination rates and higher rates of COVID-19 symptoms and experiencing hardships due to COVID-19. Final post-raking models found Hispanics had greater odds of experiencing hardships (OR = 1.81, 95% CI = 1.55, 2.11) especially among those reporting COVID-19 symptoms (OR = 2.45, 95% CI = 1.51, 3.97). The final model identified increased rates of COVID-19 vaccination among Hispanics when controlling for gender, age, and SDOH (OR = 1.22, CI = 1.09, 1.37) than have been reported nationally during the examined time period for Hispanics. Conclusions. Uptake of COVID-19 vaccination and disproportionate negative health, economic, and social impacts of COVID-19 experienced by Hispanic communities were driven by SDOH. Findings underscore the need for targeted efforts to address SDOH to achieve the best health outcomes for all.

## 1. Introduction

Hispanic individuals in the United States (U.S.) were 2, 3, and 2.3 times more likely than non-Hispanic (NH) White individuals to suffer infection, hospitalization, and death from COVID-19, respectively, from March 2020 to April 2021, prior to the release of COVID-19 vaccinations [1]. Some evidence suggests differences in frequency of reporting COVID-19 symptoms such as fever, cough, sore throat, muscles aches, with Hispanic individual citing these at higher proportions; moreover, Hispanic families have more than twice the risk of severe COVID-19 symptoms, including hospitalization, compared to other racial/ethnic groups [2,3,4]. These rates may be linked to some COVID-19 infection complication risk factors evident in Hispanic communities such as cancer, cardiovascular conditions, chronic kidney and liver disease, diabetes, HIV infection, immunocompromised condition or weakened immune system, overweight and obesity, and other conditions and factors [5,6,7]. Social and environmental factors such as lower education, lower income, substandard housing, larger household size, higher housing cost to household income ratio, higher population density, limited English proficiency, and living in counties with higher proportions of minority populations have also been associated with higher likelihood of infections and deaths [8,9,10,11]. Additionally, over 50% of Hispanic individuals work in occupations that require in-person work and proximity to others; occupational risks for COVID-19 infections have been documented in restaurants, meat and poultry processing facilities, farms, and construction settings [12,13]. Despite efforts to mitigate risks, Hispanic communities lost more individuals to premature excess deaths during the COVID-19 pandemic, at younger ages, compared to other racial/ethnic groups [14].

In March of 2021, just a few months into U.S. immunization efforts, Hispanic individuals had the lowest rate of at least one COVID-19 vaccine dose (5%) compared to White (13%), Asian (11%), and Black (7%) individuals; there has also been documented variation in vaccination rates among Hispanic individuals, depending on the state [15]. Structural factors, such as healthcare coverage, physical access to services, or availability of language-appropriate and culturally meaningful services have been documented as factors that limit access to vaccines and treatment services for COVID-19 infections [16,17]. It has also been documented that investment in trusted, community-based language-appropriate and culturally meaningful services helped close the gap in access to and uptake of COVID-19 vaccinations [18].

Social determinants of health (SDOH) play a significant role in how U.S. Hispanics access care and experienced the COVID-19 pandemic. In 2021, Hispanic individuals were 2 and 3 times more likely to be uninsured compared to Black and NH White individuals, respectively, and also less likely to have seen a healthcare provider [19,20]. The COVID-19 pandemic aggravated existing financial hardships for Hispanic individuals; they suffered higher unemployment rates and income loss, and therefore experienced difficulty paying for household expenses including rent or mortgage, compared to other racial and ethnic groups [21,22,23].

Within this context, the *All of Us* Research Program from the National Institutes of Health (NIH) provides a unique opportunity to assess the socioeconomic and health impacts of COVID-19 on U.S. Hispanics [24]. This program is inviting 1 million or more individuals across the U.S. to participate in health research to advance precision medicine, “an approach to disease treatment and prevention that seeks to maximize effectiveness by taking into account individual variability in genes, environment, and lifestyle [25].” To overcome gaps in knowledge in biomedical research, the *All of Us* Research Program has prioritized the inclusion of individuals from diverse backgrounds who are not adequately included in research [26,27].

This study assessed the relationship between SDOH (age, sex at birth, LGBT, health insurance status, housing status, education level, employment status, income) and various COVID-19 health and economic outcomes among Hispanic and NH participants in the *All of Us* Research Program. The primary aim was to characterize the multivariable SDOH and COVID-19-related profile associated with Hispanic versus non-Hispanic status in the *All of Us* research program. The primary research question was whether Hispanic and non-Hispanic participants differed in their social, economic, and COVID-19-related characteristics after simultaneous adjustment for measured covariates. We hypothesized that Hispanic participants would demonstrate a profile consistent with greater structural and pandemic-related burden. This research has been designated by the NIH as a Driver Project that will go beyond validation and result in novel findings that will drive the mission of *All of Us* and foster other novel findings and research. This study will serve as a methodology model by demonstrating the use of survey weighting via iterative proportional fitting to address representativeness limitations in large, non-probability biomedical datasets such as *All of Us*, enabling more accurate estimation of health inequities.

## 2. Methods

All data in the *All of Us* Research Workbench are mapped to the Observational Medicines Outcomes Partnership (OMOP) common data model v5.2. To protect participant privacy, a series of data transformations are applied, including data suppression of codes with a high risk of identification such as military status; generalization of categories, including age, sex at birth, gender identity, sexual orientation, and race; and date shifting by a random (less than one year) number of days, implemented consistently across each participant record. As determined by the *All of Us* Institutional Review Board, activities utilizing the Controlled Tier do not constitute research involving human subjects, and Tier and individual projects utilizing controlled tier data access do not require further IRB review. Technical details about the database and software used are provided in the online Supplemental Materials, Section S1.

### 2.1. Measures

The current analysis utilized data from the *All of Us* Researcher Workbench Version 7 data release and focused on participants’ characteristics data from two sources: “The Basics” survey and the “COVID-19 Participant Experience (COPE)” survey [28]. The Basics survey includes basic demographic, socioeconomic, and health insurance questions. The COPE survey was distributed to participants at various stages of the COVID-19 pandemic, allowing individuals to respond multiple times if they completed more than one survey. While most of the COPE survey content remained consistent across versions, some questions were altered. The survey aimed to gather data on the effects of COVID-19 on the mental and physical health of participants. The resulting data from these surveys was collected from May 2020–July 2020, November 2020, December 2020, and February 2021. For this analysis, only responses from the most recent survey from February 2021 were utilized to capture the cumulative COVID-19 outcomes related to the effects of the pandemic on everyday life as well as participants who had received a COVID-19 vaccination. Our study sample of 49,966 participants included cohorts of adults 18 years of age and older, either Hispanic (6.9%) or NH (93.1%), enrolled in the *All of Us* Research Program.

A comprehensive description of the variables utilized in this study is provided in Table S4, located in Section S3 of the Supplemental Materials. In all logistic regression models, Hispanic ethnicity (Hispanic vs. non-Hispanic) was specified as the dependent variable. SDOH variables (age, sex at birth, gender identity, sexual orientation, education, employment, annual household income, health insurance, home ownership, and stable housing status) and COVID-19-related variables (vaccination status, testing, reported symptoms, being affected by COVID-19, and experiencing economic hardship) served as independent covariates. Two multiple-response survey items, one measuring COVID-19-related impacts (e.g., changes in work setup or hours, reduction in income, or loss of employment) and one measuring pandemic-related economic hardships (e.g., difficulty paying for rent, food, medications, or other household expenses), were each coded as binary variables (1 = at least one condition endorsed; 0 = no conditions endorsed). This approach reflects the primary research interest in whether any impact or hardship was experienced and avoids overparameterization from the large number of possible response combinations; although some information about the number or severity of impacts is not captured, it provides a parsimonious method for assessing overall pandemic-related burden across a large and diverse study population.

### 2.2. Statistical Analysis

Descriptive characteristics of the participants and univariate analysis by ethnicity were conducted for all sociodemographic and COVID-19-related outcomes, using the Wilcoxon rank sum test for continuous variables and Pearson’s Chi-squared test for categorical variables. As a non-probability volunteer cohort, the All of Us Research Program does not provide pre-specified survey weights. Preliminary analysis of the data revealed disproportionate distributions for key demographic variables relative to national estimates, confirming that the unweighted sample was not representative of the U.S. population and that calibration weighting was necessary for valid population-level inference. Following data validation and imputation, iterative proportional fitting (raking) was therefore applied to create calibrated weights by iteratively adjusting participant weights until the weighted marginal distributions of age group, sex at birth, ethnicity, gender identity, sexual orientation, and employment status aligned with U.S. Census Bureau population estimates. Raking enforces agreement between weighted sample margins and population totals across all control variables simultaneously without requiring stable joint cell counts and is the standard calibration approach for non-probability samples of this structure (full technical details in Supplementary Section S1) [29,30,31]. Both unweighted and survey-weighted multivariable logistic regression models were constructed with Hispanic ethnicity as the binary dependent variable (Hispanic = 1, NH = 0) and SDOH and COVID-19 variables as predictors to assess associations. This specification was selected as a descriptive analytic approach to simultaneously characterize multivariable differences between Hispanic and non-Hispanic participants. Under this framework, odds ratios represent adjusted associations between each characteristic and membership in the Hispanic versus non-Hispanic group, conditional on all other variables in the model. The analysis was not intended to identify determinants of ethnicity or imply causal relationships. Due to a small subgroup size, sexual and gender minority populations were grouped together in the final models as LGBT. Race was removed from the final models because classification of ethnicity (Hispanic vs. NH) was observed to be highly confounded by racial identity. The very high level of correlation between race and ethnicity in the *All of Us* program may reflect the program’s use of a single multi-select item to capture both constructs. Because respondents are asked to select all categories that describe them with both race and ethnic options, a very high proportion of Hispanic respondents only selected “Hispanic” and did not select a racial identity, leading them to be grouped as “other” for the variable of race. After identifying a significant interaction between COVID-19 illness and ethnicity, subpopulation analysis for research participants with and without COVID-19-related illness was conducted to understand how Hispanic populations may have been differentially affected by COVID-19 illness with respect to economic and social indicators. Model-based results were summarized graphically using forest plots with interval estimates. All reported statistical tests indicate pairwise and multiplicity-corrected significance.

## 3. Results

The descriptive characteristics of the participants by ethnicity are presented in Table 1 for the imputed but non-survey weighted data. The demographic makeup of the *All of Us* Cohort used for this analysis deviates from national estimates for some characteristics and, as such, iterative proportional fitting (raking) was used to produce the final models presented at the end of this section. Participants predominantly identified as NH (93.1%), with Hispanics comprising only 6.9% in the non-weighted survey data. Average participant age was 60 years (SD = 15), with a median of 63 years (IQR = 21), and was largely distributed in the 25–64 (51%) and 65+ (47%) age groups. Women represented 65% of the sample, men 35%, and gender minorities 0.9%, with most participants (91%) identifying as “Straight”. Racial demographics were primarily White (83%), followed by Black (6.90%), Asian (2.4%), and others. A significant proportion of participants held an advanced degree (37%) or were college graduates (30%), with 49% employed and 38% retired. Income varied, with 34% earning between $25k and $75k annually, 13% earning below $25k, 42% earning between $75k and $200k, and 11% earning above $200k. The vast majority (98%) had health insurance and 72% owned their homes. Concerns about stable housing were reported by 7.3% of participants.

Supplementary Table S1 presents the unweighted and raked sample distributions for the five calibration variables alongside U.S. Census Bureau target estimates. Raking corrected several distributional imbalances in the unweighted sample. Most importantly, older adults (65+) were largely overrepresented (47.0% vs. the Census target of 22.0%), while younger adults (18–24) were severely underrepresented (1.6% vs. 12.0%). Hispanic participants were similarly underrepresented (6.9% vs. 18.9%), female participants were overrepresented (64.7% vs. 51.6%), and retired participants were overrepresented (37.8% vs. 20.9%). After raking, the weighted sample margins aligned with the Census targets across all five variables.

The study assessed the SDOH of 49,996 participants, distributed between Hispanic (N = 3449) and NH or Latino (N = 46,517) groups, to explore differential health outcomes during the pandemic. The Hispanic participants were generally younger with a mean age of 51 years (SD = 15) compared to NH participants who averaged 61 years (SD = 15). A substantial proportion of Hispanic participants fell within the 25–64 age group (77%), whereas NH participants were more evenly distributed across the 25–64 and 65+ age groups. Hispanic participants had higher female representation (72%) than their NH counterparts (64%). A striking difference was found in self-identified race with 76% of Hispanic participants self-identified under ‘Other’ compared to just 0.4% in the NH group, who were predominantly White (88%).

Hispanics demonstrated lower educational attainment levels, with 11% never graduating high school, more than 10 times the rate among NH participants (0.7%). Employment rates were higher among Hispanics (58% vs. 48% for NH individuals), and they also reported higher levels of housing instability and lower health insurance coverage than the NH group.

Regarding COVID-19-related variables (Section S3: Table S2 in Supplemental Materials), Hispanic participants were less likely to have received a COVID-19 vaccination (32% vs. 44%, *p* < 0.001) but more likely to have been tested for COVID-19 (59% vs. 41%, *p* < 0.001). Compared to NH participants, a higher percentage of Hispanics reported being sick with COVID-19 symptoms (7% vs. 3.5%, *p* < 0.001), being directly affected by COVID-19 (66% vs. 52%, *p* < 0.001), and experiencing economic hardships due to COVID-19 (22% vs. 6.5%, *p* < 0.001).

### Model-Based Results

**Figure 1:**
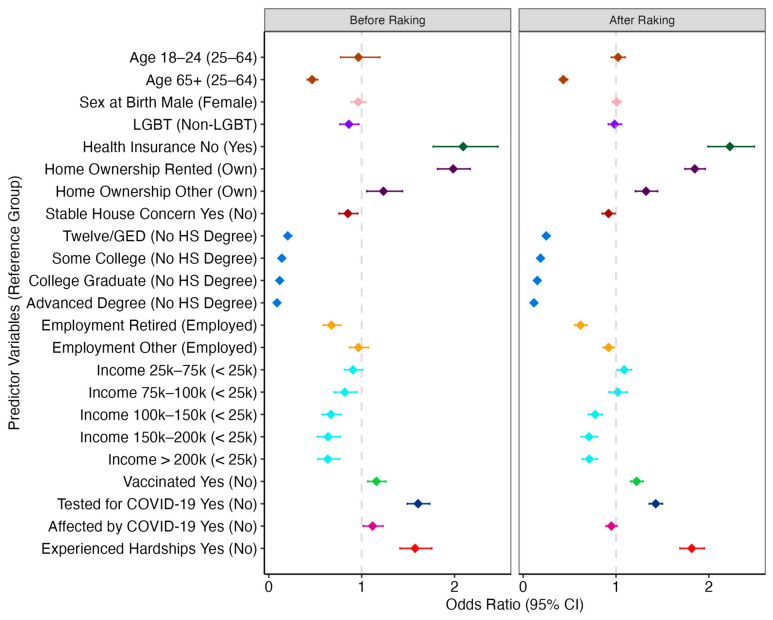
Forest plots of multivariable logistic regression models examining adjusted associations between social and environmental factors of health and Hispanic versus non-Hispanic status for all study participants.

**Figure 2:**
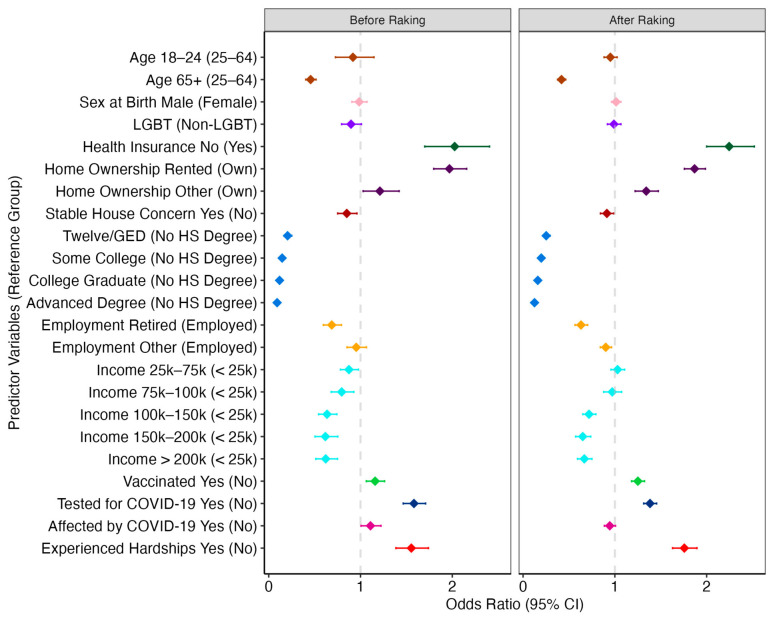
Forest plots of multivariable logistic regression models examining adjusted associations between social and environmental factors of health and Hispanic versus non-Hispanic status for participants without COVID-19 illness.

**Figure 3:**
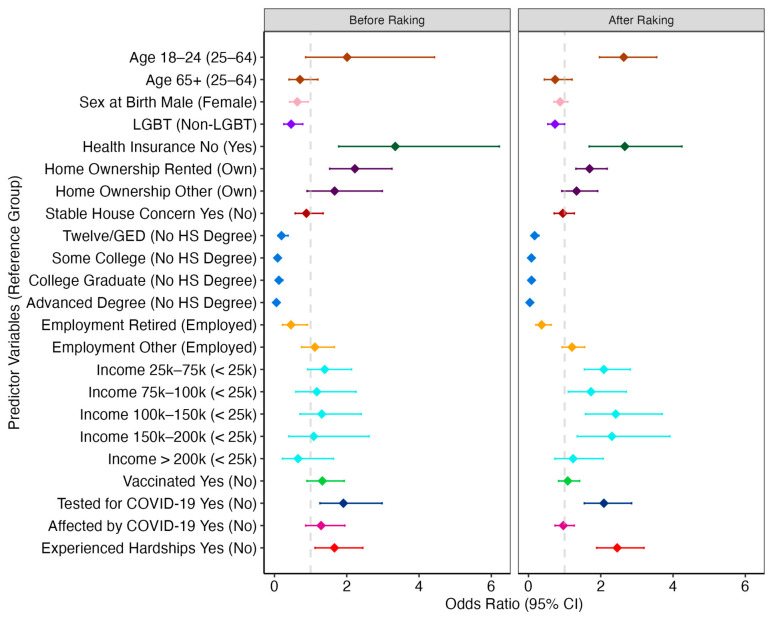
Forest plots of multivariable logistic regression models examining adjusted associations between social and environmental factors of health and Hispanic versus non-Hispanic status for participants with COVID-19 illness.

Results from six multivariable logistic regression models modeling associations between being Hispanic and the health outcomes are presented in a forest plot in Figure 1, Figure 2 and Figure 3. For the models, sexual minorities and gender minorities are grouped together under the label LGBT. Race was dropped because classification of ethnicity (Hispanic vs. NH) was observed to be highly confounded by racial identity. Full results of the logistic regression models with individual odds ratios associating being Hispanic with social and environmental factors of health can be found in the online Supplemental Materials, Sections S2 and S3, for both pre- and post-raking data.

After raking, the odds of being 65 years and older for Hispanic populations was 57% lower compared to 25–64 years old (OR = 0.43, 95% CI = 0.36, 0.51). There were no significant differences in the odds of being 18–24 years old, male (sex assigned at birth), or identifying as LGBT for Hispanic versus NH populations. Hispanics had 85% higher odds of renting (OR = 1.85, 95% CI = 1.64, 2.09) and 32% higher odds of other housing arrangements (OR = 1.32, 95% CI = 1.03, 1.69) than NH populations. However, among Hispanics, there were no differences in the odds of experiencing stable housing concerns versus NH populations.

For educational attainment, Hispanic participants had lower odds of completing high school or GED (OR = 0.25, 95% CI = 0.19, 0.33), some college (OR = 0.19, 95% CI = 0.14, 0.24), being a college graduate (OR = 0.15, 95% CI = 0.12, 0.20), or completing an advanced degree (OR = 0.12, 95% CI = 0.09, 0.15) when compared to NH participants. Being Hispanic lowered the odds of being retired by 38% (OR = 0.62, 95% CI = 0.52, 0.73). There were no significant differences among $25,000–$75,000 or $75,000–$100,000 in annual household income compared to making less than $25,000 among Hispanic and NH populations; however, Hispanic participants had lower odds of the higher income levels including $100,000–$150,000 (OR = 0.78, 95% CI= 0.62, 0.96), $150,000–$200,000 (OR = 0.70, 95% CI = 0.54, 0.91), and more than $200,000 (OR = 0.71, 95% CI = 0.54, 0.94).

Hispanic respondents had over twice the odds of not having health insurance (OR = 2.23, 95% CI = 1.74, 2.86) and 43% higher odds of reporting being tested for COVID-19 than NH populations (OR = 1.43, 95% CI = 1.28, 1.59). Among Hispanic participants, after controlling for SDOH variables (age, sex at birth, LGBT, health insurance status, housing status, education level, employment status, income), there were increased odds of reporting they had received a COVID-19 vaccination (OR = 1.22, 95% CI = 1.09, 1.37) than NH populations. There was no significant difference in the odds of being affected by COVID-19 for Hispanic and NH populations, but Hispanic participants had 81% higher odds of having experienced economic hardship due to COVID-19 (OR = 1.81, 95% CI = 1.55, 2.11). Moreover, Hispanic participants who reported COVID-19 illness also reported more than twice the odds of experiencing hardship (OR = 2.45, 95% CI = 1.51, 3.97) than NH populations.

Comparison of pre- and post-raking estimates revealed that raking altered both the magnitude and statistical significance of several associations. For instance, before raking, Hispanic status was significantly associated with being affected by COVID-19 (OR = 1.12, 95% CI = 1.02–1.23, *p* = 0.022); this association was no longer statistically significant after raking, indicating it was partly attributable to demographic non-representativeness in the unweighted sample. In contrast, the association between Hispanic status and economic hardship persisted and strengthened after raking (pre-raking OR = 1.57, 95% CI = 1.41–1.75; post-raking OR = 1.81, 95% CI = 1.55–2.11), affirming the robustness of this disparity to sample reweighting. Post-raking estimates are reported as the primary findings given their population-representative nature.

## 4. Discussion

These results provide further evidence that U.S. Hispanics were disproportionately affected by the COVID-19 pandemic, when compared to their NH counterparts. In all population-adjusted models, Hispanics reported higher rates of experiencing health impacts and economic hardship due to COVID-19.

The proportion of Hispanic participants in the *All of Us* participant cohort who received COVID-19 vaccination was 32%, as documented in the February 2021 version of the COPE survey, and was lower than that of NH Whites (44%). Although the trend is similar, the overall immunization rates in the *All of Us* Research Program were much higher than reported in the KFF which found that in March 2021, 5% and 13% of Hispanic and NH White individuals in the U.S., respectively, had received at least one COVID-19 vaccine [15]. Differences in reporting vaccination proportions have consistently varied based on a variety of factors; for example, both KFF and APM Research Lab analyses have shown differences in vaccination proportions in Hispanic individuals by state [15,32].

We note too that although univariate analysis demonstrated lower vaccination rates for Hispanic vs. non-Hispanic populations in the *All of Us* database, the multivariate logistic regression model results indicate higher vaccination rates for Hispanics. Though seemingly contradictory, the model-based results control for factors known to impact vaccination status, such as income, gender, age and others, and affect the adjusted association between being Hispanic and vaccinated. This apparent change in effect suggests that Hispanic identity itself is not associated with lower vaccine uptake but instead the other structural and systemic SDOH that may help explain reported lower vaccination rates. A similar trend was observed by KFF data that observed by February 2023, with concerted efforts to increase vaccination access, Hispanic individuals had higher proportions of having received at least dose of the COVID-19 vaccine compared to non-Hispanic individuals (67% versus 57%) [33].

Similar to 2021 U.S. Census Bureau data, the proportion of uninsured Hispanic *All of Us* participants was five times that of NH White participants (7.7% versus 1.5%). Of *All of Us* participants who identify as Hispanic, 14% expressed concern for stable housing compared to just under 7% for NH participants in the February 2021 COPE survey; this is similar to Household Pulse Survey for 3–15 February 2021, where 16% of Hispanic respondents indicated that they had no confidence that they would be able to pay their next month’s rent compared to 7% NH White respondents [19,23].

In a nationally representative sample, Hair and Urban found that even moderate, mild, or asymptomatic COVID-19 can increase the odds of lost earnings by 21%, which can more than double for those with severe or persistent COVID-19 symptoms [4]. This effect was more pronounced among individuals below 200% of the poverty line where moderate, mild, or asymptomatic COVID-19 can increase the odds of lost earnings by 86% and quadruple the odds for those with severe or persistent COVID-19 symptoms [4]. Similarly, in our study, the odds ratio of reporting COVID-19 symptoms to experiencing financial hardships was 2.45 (95% CI = 1.51, 3.97) among Hispanic *All of Us* participants, demonstrating an increased impact on Hispanic communities.

The comprehensive analysis of sociodemographic and COVID-19-related variables across Hispanic and NH participants within the study reveals statistically significant differences (*p* < 0.001) in all examined aspects within the *All of Us* participant population. While other studies cite similar findings, we note that this study presents implications to health outcomes for Hispanic populations that other studies did not have the data to support [4]. In particular, it is clear in the analysis that Hispanic communities experienced more personal hardships and economic impacts during the COVID-19 pandemic. These impacts remained statistically significant even when controlling for major participant-level characteristics, such as age, income, and sex. This finding reinforces evidence that structural and social determinants of health play a central role in shaping inequitable pandemic outcomes among Hispanic populations [8,9,11,16,21,22]. This implies that during major health events, such as a worldwide pandemic, efforts to mitigate personal and economic hardships are important to mitigate negative impacts.

Several limitations arose during the process of analysis. The *All of Us* Research Program did not include children at the time of our analysis, so there was no representation of children in this study. Although one-quarter of the child population in the U.S. is Hispanic, the differences in Hispanic children and NH children could not be studied in the *All of Us* workspace for this reason [34]. *All of Us* is addressing this limitation by currently pilot-testing a pediatric enrollment program [35]. Another limitation is that the *All of Us* Research Program does not allow researchers to have access to the specific subpopulations of Hispanics in the U.S. or place of origin. U.S. Hispanics are not a monolithic group, and subgroups may experience very different health disparities [36,37]. Additionally, COVID-19-related outcomes and social determinant measures were self-reported and may therefore be subject to recall bias, social desirability bias, misclassification, or incomplete reporting. In addition, although raking was used to improve alignment with population margins, the All of Us Research Program is a non-probability volunteer cohort rather than a population-based probability sample. As a result, findings should be interpreted as adjusted associations within the All of Us COPE survey population, and caution is warranted when extrapolating these results to the broader U.S. Hispanic population.

Lastly, when looking at the population margins for other variables such as race, biological sex, gender identity, age, and sexual orientation, there were some limitations in adequate U.S. population reference distributions. For example, there was no census population data available for the non-binary category in the Gender Identity variable, which the *All of Us* program does collect. Although recently updated for future collection of race and ethnicity data, available population-level statistics from the U.S. Census Bureau were not as inclusive as the data collected and reported for *All of Us*. Further, due to the small number of gender minorities, sexual and gender minorities were grouped together for regression analyses to ensure estimable models. While sexual minorities and gender minorities may have distinct experiences within the healthcare system and our society, they may also have shared experiences of stigma and prejudice that can affect health outcomes.

As this was designated by the NIH as a Driver Project for *All of Us*, we note several important findings for other researchers utilizing the *All of Us* Research Workbench. Despite comprising over 19% of total participants in the *All of Us* Research Program at the time of the Version 7 data release, Hispanics made up only 6.9% in the non-weighted survey data, indicating significant barriers to ongoing engagement and retention in the program [38]. NIH is aware of these differences in engagement and retention and has recently begun efforts to address them [39]. In the meantime, the iterative proportional fitting (raking) utilized in this driver project (described in detail in Section S1) provides a reasonable approach for addressing representativeness datasets for underrepresented groups in biomedical research. This approach demonstrates how statistical weighting techniques can be used to mitigate biases introduced by non-representative samples and enhance the validity of health inequities research. Comparing unweighted and raked model estimates showed that correcting for the sample’s demographic imbalances changed some findings in substantive ways. Most notably, the association between Hispanic status and being broadly affected by COVID-19 was statistically significant in the unweighted analysis but not after raking, suggesting it was partially driven by the overrepresentation of certain demographic groups in the COPE survey rather than a true population-level relationship. Findings that held after raking, particularly the association with economic hardship, are therefore more likely to reflect genuine disparities experienced by Hispanic communities. More broadly, our findings underscore the importance of combining methodological rigor with inclusive data collection strategies to ensure that analyses of large-scale biomedical datasets accurately reflect the populations most affected by structural inequities.

## 5. Conclusions

In conclusion, our findings underscore the disproportionate impact of COVID-19 on Hispanic communities. Among *All of Us* participants, Hispanics reported worse outcomes across all COVID-19-related variables: lower vaccination rates and higher rates of COVID-19 symptoms, testing, being affected by COVID-19, and experiencing hardships due to COVID-19 compared to NH *All of Us* participants. Even with post-raking, the final models consistently demonstrated greater overall pandemic-related hardships reported by Hispanic participants of the *All of Us* Research Program. The final models also demonstrated that COVID-19 Hispanic vaccination rates, during the period examined, after controlling for SDOH were higher than national data were showing. Public health strategies today and during future pandemics must focus on mitigating these impacts by providing resources to trusted providers of information and services and offering language-accessible and culturally meaningful interventions to support Hispanic communities, specifically those who are most affected by health and economic impacts. Social determinants of health were shown to be a significant factor in vaccination rates for Hispanic communities and addressing them should be central to any health effort, including public health emergency response. Efforts should aim to improve access to healthcare, including vaccinations, and economic support, particularly during major health crises. Recognizing the unique needs and experiences within diverse Hispanic subgroups is crucial for effective intervention. Addressing community-defined needs and delivering tailored responses is vital for ensuring the best health outcomes in communities.

## Figures and Tables

**Table 1:** Sociodemographic characteristics of study participants (N = 49,966).

Characteristic	Total	Ethnicity		
	N = 49,966 ^1^	Hispanic, N = 3449 (6.9%) ^1^	Not Hispanic, N = 46,517 (93.1%) ^1^	*p*-Value ^2^
Age				<0.001
Mean (SD)	60 (15)	51 (15)	61 (15)	
Median (IQR)	63 (21)	51 (23)	64 (20)	
Age Group				<0.001
25–64	25,680 (51%)	2660 (77%)	23,020 (49%)	
18–24	790 (1.6%)	109 (3.2%)	681 (1.5%)	
65+	23,496 (47%)	680 (20%)	22,816 (49%)	
Sex at Birth				<0.001
Female	32,304 (65%)	2470 (72%)	29,834 (64%)	
Male	17,662 (35%)	979 (28%)	16,683 (36%)	
Race				<0.001
White	41,684 (83%)	653 (19%)	41,031 (88%)	
Black or African American	3450 (6.9%)	57 (1.7%)	3393 (7.3%)	
Asian	1212 (2.4%)	26 (0.8%)	1186 (2.5%)	
More than one population	816 (1.6%)	81 (2.3%)	735 (1.6%)	
Other	2804 (5.6%)	2632 (76%)	172 (0.4%)	
Gender Identity				<0.001
Woman	31,978 (64%)	2433 (71%)	29,545 (64%)	
Man	17,553 (35%)	972 (28%)	16,581 (36%)	
Gender Minority	435 (0.9%)	44 (1.3%)	391 (0.8%)	
Sexual Orientation				<0.001
Straight	45,444 (91%)	3049 (88%)	42,395 (91%)	
Sexual Minority	4522 (9.1%)	400 (12%)	4122 (8.9%)	
Highest Education				<0.001
Never Graduated High School	678 (1.4%)	364 (11%)	314 (0.7%)	
Twelve Or GED	3919 (7.8%)	499 (14%)	3420 (7.4%)	
Some College	11,571 (23%)	945 (27%)	10,626 (23%)	
College Graduate	15,209 (30%)	925 (27%)	14,284 (31%)	
Advanced Degree	18,589 (37%)	716 (21%)	17,873 (38%)	
Employment Status				<0.001
Employed	24,541 (49%)	2000 (58%)	22,541 (48%)	
Retired	18,876 (38%)	524 (15%)	18,352 (39%)	
Other	6549 (13%)	925 (27%)	5624 (12%)	
Annual Household Income				<0.001
Less than 25k	6461 (13%)	1038 (30%)	5423 (12%)	
25k–75k	17,223 (34%)	1280 (37%)	15,943 (34%)	
75k–100k	7265 (15%)	370 (11%)	6895 (15%)	
100k–150k	9328 (19%)	387 (11%)	8941 (19%)	
150k–200k	4259 (8.5%)	167 (4.8%)	4092 (8.8%)	
More than 200k	5430 (11%)	207 (6.0%)	5223 (11%)	
Health Insurance				<0.001
Yes	49,001 (98%)	3183 (92%)	45,818 (98%)	
No	965 (1.9%)	266 (7.7%)	699 (1.5%)	
Home Ownership				<0.001
Own	35,776 (72%)	1490 (43%)	34,286 (74%)	
Rent	11,593 (23%)	1701 (49%)	9892 (21%)	
Other Arrangement	2597 (5.2%)	258 (7.5%)	2339 (5.0%)	
Stable House Concern				<0.001
No	46,304 (93%)	2951 (86%)	43,353 (93%)	
Yes	3662 (7.3%)	498 (14%)	3164 (6.8%)	

^1^ *n* (%); ^2^ Wilcoxon rank sum test; Pearson’s Chi-squared test.

## Data Availability

The data used in this study are available through the NIH All of Us Research Program Researcher Workbench. Access requires registration and approval.

## Supplementary Materials

The following supporting information can be downloaded at: https://www.mdpi.com/article/10.3390/ijerph23070859/s1, Table S1: Distribution of Variables Used in Raking; Table S2: COVID-19-Related Variables by Ethnicity; Table S3: Ordinary and survey-weighted multivariable logistic regression models examining adjusted associations between social and environmental factors of health and Hispanic versus non-Hispanic status (Hispanic status as dependent variable); Table S4: Post-raking multivariable logistic regression models examining adjusted associations between social and environmental factors of health and Hispanic versus non-Hispanic status for study participants with and without COVID-19-related illness (Hispanic status as dependent variable); Table S5: Description of variables used in the study [29,30,31,40,41,42,43,44,45,46,47,48,49,50,51,52,53].
