# Supplementary material for: Utilizing the All of Us Dataset to Assess the Socioeconomic and Health Impacts of COVID-19 on Hispanics in the United States
Source: Int J Environ Res Public Health. 2026 Jun 30;23(7):859. doi: 10.3390/ijerph23070859 (PMC13409778; doi:10.3390/ijerph23070859)
Supplement: Supplementary file 1 [file ijerph-23-00859-s001.zip › ijerph-4294054-supplementary.pdf]

Supplemental Materials:

Utilizing the *All of Us* Dataset to Assess the Socioeconomic and Health Impacts of COVID-19 on Hispanics in the United States

SECTION S1: TECHNICAL DESCRIPTION OF DATA ANALYSIS SOFTWARE, DATA PROCESSING, AND MODEL FITTING 2

DATA PROCESSING 2

REFERENCES 3

SECTION S2: PRE-RAKING AND POST-RAKING RESULTS OF LOGISTIC REGRESSION MODELS WITH INDIVIDUAL ODDS RATIOS PREDICTING HISPANIC STATUS AS A FUNCTION OF SOCIAL AND ENVIRONMENTAL FACTORS 5

SECTION S3: TABLES OF VARIABLES, DISTRIBUTION OF VARIABLES USED IN RAKING AND LOGISTIC REGRESSION MODELS WITH INDIVIDUAL ODDS RATIOS PREDICTING HISPANIC STATUS AS A FUNCTION OF SOCIAL AND ENVIRONMENTAL FACTORS 9

TABLE S1: DISTRIBUTION OF VARIABLES USED IN RAKING 9

TABLE S2: COVID-19-RELATED VARIABLES BY ETHNICITY 10

TABLE S3: ORDINARY AND SURVEY-WEIGHTED MULTIPLE LOGISTIC REGRESSION MODELS PREDICTING HISPANIC STATUS AS A FUNCTION OF SOCIAL AND ENVIRONMENTAL FACTORS OF HEALTH. 11

TABLE S4: POST-RAKING LOGISTIC REGRESSION MODELS PREDICTING HISPANIC STATUS AS A FUNCTION OF SOCIAL AND ENVIRONMENTAL FACTORS OF HEALTH. 14

TABLE S5: DESCRIPTION OF VARIABLES USED IN THE STUDY. 17

Section S1: Technical Description of Data Analysis Software, Data Processing, and Model Fitting

Documentation on privacy implementation and creation of the Curated Data Repository (CDR) is available in the *All of Us* Registered Tier CDR Data Dictionary4.[1] The Researcher Workbench was used to select groups of participants (Cohort Builder), create the datasets for analysis (Dataset Builder), and analyze all data (Jupyter Notebooks). The Notebooks enabled the use of saved datasets and direct query using R and Python 3 programming languages. In all sections below, the data processing and analysis made use of both *R*[2] and *Python 3,*[3] incorporating modules *numpy*[4] *and matplotlib*[5] in *Python* and packages *gtsummary*,[6] *mice*,[7]*survey*,[8] and *ggplot*[9] in *R*.

Data Processing

In the extracted data, 12,518 participants had missing data on at least one variable arising from nonresponse (skipped questions) and explicit "Prefer not to answer" options. These variables, affected by COVID-19, annual household income, and experienced some form of hardship due to COVID-19, recorded missing data ranging from 2% to 10%. A random forest imputation procedure based on the Multivariate Imputation by Chained Equations (MICE) algorithm[7] was used to impute missing values that were deemed missing at random. Because of the high risk for non-random missing values in sex at birth, gender identity, and sexual orientation (amounting to 1,070 observations), these observations were not imputed and instead subsequently excluded from the analysis. Convergence of the imputation model was reached, and the data stored with complete observations. The plots visually confirming convergence are available upon request to the corresponding author. Iterative proportional fitting (IPF), also referred to as raking ratio estimation or raking for survey data[14-15], was used to calibrate survey weights so that the marginal distributions of selected key demographic variables (age group, sex at birth, ethnicity, gender identity, sexual orientation, and employment status) in the All of Us Basics and COPE sample aligned with corresponding US population margins extracted from the US Census Bureau data.[10-13] This became necessary after preliminary analysis of the data revealed disproportionate distributions for these variables, compared to their national estimates. Raking is a calibration-weighting method that iteratively adjusts sampling weights to satisfy known marginal totals while preserving the original sample structure[15-16]. The algorithm proceeds by sequentially scaling weights across each control variable until convergence is achieved, defined as agreement between weighted sample margins and population targets within default tolerance levels implemented in the survey R package[8]. Through this adjustment of weights, the observed data is weighted to resemble the population, reducing bias and enabling the possibility of making inferences about the entire population. Population percentages were converted to frequencies by multiplying each marginal proportion by the analytic sample size (N = 49,966) to meet the input requirements of the raking function. All raking procedures were conducted using the survey package in R.

After treating missing values by imputation and rowwise deletion, the study included 49,966 participants in exploring the disproportionate impact of COVID-19 on Hispanic populations in the U.S. A summary table showing the distribution of variables involved in the raking process pre-raking and post-raking is presented in Section S3, Table 1.

We fitted both unweighted (ordinary) and survey-weighted multivariable logistic regression models to examine associations between Hispanic status and the social and environmental determinants of health factors considered in the study. The unweighted models were estimated using standard logistic regression (glm()), assuming independent observations and equal contribution of each participant. The survey-weighted models incorporated the calibrated sampling weights derived from iterative proportional fitting and were estimated using the svyglm() function in the R survey package [8,17]. These models apply the raked weights to obtain population-representative effect estimates and use robust variance estimators. Because no clustering or stratification variables were specified in the raking design (id = ~1), weighting was the only design feature incorporated.

References

1. Framework for Access to *All of Us* Data Resources. https://www.researchallofus.org/wp-content/themes/research-hub-wordpress-theme/media/data&tools/data-access-use/AoU_Data_Access_Framework_508.pdf.
2. R Core Team (2023). _R: A Language and Environment for Statistical Computing_. R Foundation for Statistical Computing, Vienna, Austria. https://www.R-project.org/.
3. Van Rossum, G., & Drake, F. L. (2009). Python Language Reference, version 3. Available at Python Software Foundation.
4. Harris, C.R., Millman, K.J., van der Walt, S.J. et al. (2020). Array programming with NumPy. Nature, 585, 357–362. DOI: 10.1038/s41586-020-2649-2.
5. Hunter, J. D. (2007). Matplotlib: A 2D graphics environment. Computing in Science & Engineering, 9(3), 90-95.
6. Sjoberg DD, Whiting K, Curry M, Lavery JA, Larmarange J. Reproducible summary tables with the gtsummary package. The R Journal 2021;13:570–80. https://doi.org/10.32614/RJ-2021-053.
7. Stef van Buuren, Karin Groothuis-Oudshoorn (2011). mice: Multivariate Imputation by Chained Equations in R. Journal of Statistical Software, 45(3), 1-67. DOI 10.18637/jss.v045.i03.
8. T. Lumley (2023) "survey: analysis of complex survey samples". R package version 4.2.
9. H. Wickham. ggplot2: Elegant Graphics for Data Analysis. Springer-Verlag New York, 2016.

10. U.S. Census Bureau. Hispanic Heritage Month 2022. https://www.census.gov/newsroom/facts-for-features/2022/hispanic-heritage-month.html. Accessed on July 16, 2023.
11. U.S. Census Bureau. Acs Demographic and Housing Estimates. American Community Survey, ACS 1-Year Estimates Data Profiles, Table DP05, 2021, https://data.census.gov/table/ACSDP1Y2021.DP05?d=ACS 1-Year Estimates Data Profiles. Accessed on July 16, 2023.
12. U.S. Census Bureau. Sexual Orientation and Gender Identity in the Household Pulse Survey, July 21-September 13, 2021. https://www.census.gov/library/visualizations/interactive/sexual-orientation-and-gender-identity.html. Accessed on July 16, 2023.
13. U.S. Census Bureau. Selected Economic Characteristics. American Community Survey, ACS 1-Year Estimates Data Profiles, Table DP03, 2021, https://data.census.gov/table/ACSDP1Y2021.DP03?t=Employment and Labor Force Status&g=010XX00US. Accessed on July 16, 2023.
14. Deming WE, Stephan FF. On a least squares adjustment of a sampled frequency table when the expected marginal totals are known. *Ann Math Stat.* 1940;11(4):427-44. doi:10.1214/aoms/1177731829
15. Deville JC, Särndal CE. Calibration estimators in survey sampling. *J Am Stat Assoc.* 1992;87(418):376-82. doi:10.1080/01621459.1992.10475217
16. Battaglia MP, Izrael D, Hoaglin DC, Frankel MR. Practical considerations in raking survey data. *Surv Pract.* 2009;2(5). doi:10.29115/SP-2009-0015
17. Lumley T, Scott A. Fitting regression models to survey data. *Stat Sci.* 2017;32(2):265-278. doi:10.1214/16-STS605

Section S2: Pre-Raking and Post-Raking Results of Logistic Regression Models with Individual Odds Ratios Examining Adjusted Associations between Hispanic Ethnicity and Social and Environmental Factors

Study Participant Data (Pre-Raking; Section S3, Table 2)

Before raking, the odds of identifying as Hispanic and being 65 years and older were almost half compared to 25-64 years old (OR=0.47, 95% CI=0.41, 0.52; p-value<0.001). There were no significant differences in the odds of identifying as Hispanic among those who were 18-24 years old compared to 25-64 years old, or between men and women (sex assigned at birth). The odds of identifying as Hispanic among LGBT individuals were 14% less than being non-LGBT (OR=0.86, 95% CI= 0.77, 0.97; p-value=0.015).

Housing and Education: Participants who rented had almost two times the odds of identifying as Hispanic (OR=1.99, 95% CI=1.82, 2.17; p-value<0.001) and those with other housing arrangements had 23% higher odds of being Hispanic (OR=1.23, 95% CI=1.05, 1.44; p-value=0.009), compared to those owned their home; however, participants who reported stable housing concern had 15% lower odds of identifying as Hispanic (OR=0.85, 95% CI=0.76, 0.96; p-value=0.009). Participants with higher educational attainment had lower odds of being Hispanic than those who did not complete high school: 80% lower odds for those who completed high school or GED (OR=0.20, 95% CI=0.17, 0.25; p-value<0.001), 86% lower odds for those who completed some college (OR=0.14, 95% CI=0.12, 0.17; p-value<0.001), 88% lower odds for college graduates (OR=0.12, 95% CI=0.10, 0.14; p-value<0.001), and 91% lower odds for those who completed an advanced degree (OR=0.09, 95% CI=0.07, 0.11; p-value<0.001).

Employment Status and Income: Being retired lowered the odds of being Hispanic by 32% (OR=0.68, 95% CI=0.59, 0.78, p-value<0.001). There were no significant differences in Hispanic status and making $25,000-75,000 in annual household income compared to making less than $25,000; however, participants had lower odds of being Hispanic if making $75,000 and more, compared to less than $25,000: 18% lower odds for those making $75,000-100,000 (OR=0.82, 95% CI=0.70, 0.95; p-value= 0.009), 33% lower odds for those making $100,000-150,000 (OR=0.67, 95% CI= 0.58, 0.78; p-value<0.001), 36% lower odds for those making $150,000-200,000 (OR=0.64, 95% CI=0.52, 0.77; p-value<0.001), and 37% lower odds for those earning more than $200,000 (OR=0.63, 95% CI=0.53, 0.76; p-value<0.001).

Access to Care: Participants who did not to have health insurance had over twice the odds of identifying as Hispanic (OR=2.10, 95% CI=1.77, 2.47; p-value<0.001), but having received COVID-19 vaccination increased the odds of being Hispanic by 16% (OR=1.16, 95% CI=1.07, 1.26; p-value<0.001) and being tested for COVID-19 increased the odds by 61% (OR=1.61, 95% CI=1.49, 1.73; p-value<0.001).

Affected by COVID-19: Participants who reported being affected by COVID-19 had 12% higher odds of identifying as Hispanic (OR=1.12, 95% CI=1.02, 1.23; p-value=0.022) and having experienced hardship due to COVID-19 increased the odds by 57% (OR=1.57, 95% CI=1.41, 1.75; p-value<0.001).

Study Participant Data (Post-Raking; Section S3, Table 2)

After raking, the odds of identifying as Hispanic and being 65 years and older were 57% lower compared to 25-64 years old (OR=0.43, 95% CI=0.36, 0.51; p-value<0.001). There were no significant differences in the Hispanic status for participants who were 18-24 years old compared to 25-64 years old, between men and women (sex assigned at birth), or between LGBT and non-LGBT individuals.

Housing and Education: Participants who rented and those with other housing arrangements, respectively, had 85% (OR=1.85, 95% CI=1.64, 2.09; p-value<0.001) and 32% (OR=1.32, 95% CI=1.03, 1.69; p-value=0.031) higher odds of being Hispanic compared to those who owned their home. After raking, there were no differences in the Hispanic status and experiencing stable housing concerns. Participants with higher educational attainment had lower odds of being Hispanic than those who did not complete high school: 75% lower odds for those who completed high school or GED (OR=0.25, 95% CI=0.19, 0.33; p-value<0.001), 81% lower odds for those who completed some college (OR=0.19, 95% CI=0.14, 0.24; p-value<0.001), 85% lower odds for college graduates (OR=0.15, 95% CI=0.12, 0.20; p-value<0.001), and 88% lower odds for those who completed an advanced degree (OR=0.12, 95% CI=0.09, 0.15; p-value<0.001).

Employment Status and Income: Being retired lowered the odds of identifying as Hispanic by 38% (OR=0.62, 95% CI=0.52, 0.73, p-value<0.001). There were no significant differences in Hispanic status for those making $25,000-75,000 or $75,000-100,000 in annual household income compared to individuals making less than $25,000; however, compared to those making less than $25,000, those making $100,000 and more had lower odds of being Hispanic: 22% lower odds for those making $100,000-150,000 (OR=0.78, 95% CI= 0.62, 0.96; p-value=0.022), 30% lower odds for those making $150,000-200,000 (OR=0.70, 95% CI=0.54, 0.91; p-value=0.008), and 29% lower odds for those earning more than $200,000 (OR=0.71, 95% CI=0.54, 0.94; p-value=0.016).

Access to Care: Those reporting not having health insurance had over twice the odds of identifying as Hispanic (OR=2.23, 95% CI=1.74, 2.86; p-value <0.001), but having received a COVID-19 vaccination increased the odds of being Hispanic by 22% (OR=1.22, 95% CI=1.09, 1.37; p-value<0.001) and having been tested for COVID-19 increased the odds by 43% (OR=1.43, 95% CI=1.28, 1.59; p-value<0.001).

Affected by COVID-19: After raking, there was no significant difference in the odds being Hispanic and reports of being affected by COVID-19, but those who reported hardship due to COVID-19 had 81% higher odds of being Hispanic (OR=1.81, 95% CI=1.55, 2.11; p-value<0.001).

Subpopulation analysis: study participants who did not report COVID-19 illness (Post-Raking; Section S3, Table 3)

For Hispanic participants without COVID-19 illness, the odds of identifying as Hispanic and being 65 years and older were 58% lower compared to individuals of 25-64 years of age (OR=0.42, 95% CI=0.35, 0.50; p-value<0.001). There were no significant differences in the odds of being Hispanic for individuals of 18-24 years of age compared to 25-64 years old,

between men and women (sex assigned at birth), or between LGBT and non-LGBT individuals. Housing and Education: Participants who rented their home had 87% higher odds (OR=1.87, 95% CI=1.66, 2.11; p-value<0.001), and those who had other arrangements had 33% higher odds (OR=1.33, 95% CI=1.03, 1.73; p-value=0.028) of identifying as Hispanic, compared to those who owned their home. After raking, there were no differences in the odds of being Hispanic and experiencing stable housing concerns for participants without COVID-19 illness. Participants who did not report COVID-19 illness and who had higher educational attainment, had lower odds of being Hispanic than those who did not complete high school: 75% lower odds for those who completed high school or GED (OR=0.25, 95% CI=0.19, 0.33; p-value<0.001), 80% lower odds those who completed some college (OR=0.20, 95% CI=0.15, 0.26; p-value<0.001), 84% lower odds for college graduates (OR=0.16, 95% CI=0.12, 0.21; p-value<0.001), and 88% lower odds those who completed an advanced degree (OR=0.12, 95% CI=0.09, 0.16; p-value<0.001).

Employment Status and Income: Being retired lowered the odds of being Hispanic by 37% lower (OR=0.63, 95% CI=0.53, 0.75, p-value<0.001). There were no significant differences in Hispanic status for those making $25,000-75,000 or $75,000-100,000 in annual household income compared to making less than $25,000; however, participants making $100,000 and more had lower odds of identifying as Hispanic compared to those making less than $25,000: 28% lower odds for those making $100,000-150,000 (OR=0.72, 95% CI= 0.57, 0.90; p-value=0.004), 35% lower odds for those making $150,000-200,000 (OR=0.65, 95% CI=0.50, 0.83; p-value <0.001), and 33% lower odds for those earning more than $200,000 (OR=0.67, 95% CI=0.51, 0.87; p-value=0.003).

Access to Care: Study participants without health insurance had over twice the odds of identifying as Hispanic (OR=2.25, 95% CI=1.72, 2.93; p-value<0.001), while those who reported having received COVID-19 vaccination had 25% higher odds (OR=1.25, 95% CI=1.11, 1.41; p-value<0.001) and those who tested for COVID-19 had 38% higher odds of Hispanic status (OR=1.38, 95% CI=1.24, 1.54; p-value<0.001).

Affected by COVID-19: After raking, there was no significant difference in Hispanic status and being affected by COVID-19, but participants who reported hardship due to COVID-19 had 75% higher odds of indicating that they were Hispanic (OR=1.75, 95% CI=1.49, 2.06; p-value<0.001).

Subpopulation analysis: Study participants who reported COVID-19 illness (Post-Raking; Section S3, Table 3)

For Hispanic participants with COVID-19 illness, the odds of identifying as Hispanic and being 65 years and older were not significantly different compared to 25-64 years old. However, the odds of identifying as Hispanic were 2.63 times higher for those who were18-24 years old compared to 25-64 years old (OR=2.63, 95% CI=1.13, 6.15; p-value<0.025). There were no significant differences in the odds of identifying Hispanic between men and women (sex assigned at birth), or between LGBT and non-LGBT individuals.

Housing and Education: Those who rented their home had increased the odds of identifying as Hispanic by 69% compared to those who own their home (OR=1.69, 95% CI=1.04, 2.76; p-value=0.034); however, for those with COVID-19 illness, there were no significant differences in

Hispanic status for those who had other arrangements compared to those who owned their home. After raking, there were no differences in Hispanic status and experiencing stable housing concerns for participants with COVID-19 illness. Participants with COVID-19 illness, and who higher educational attainment, had lower odds of being Hispanic than those who did not complete high school: 83% lower odds for those who completed high school or GED (OR=0.17, 95% CI=0.08, 0.37; p-value<0.001), 92% lower odds for those who completed some college (OR=0.08, 95% CI=0.04, 0.16; p-value<0.001), 92% lower odds for those who were college graduates (OR=0.08, 95% CI=0.04, 0.18; p-value<0.001), and 96% lower odds for those who completed an advanced degree (OR=0.04, 95% CI=0.02, 0.09; p-value<0.001).

Employment Status and Income: For those with COVID-19 illness, being retired lowered the odds of identifying as Hispanic by 64% (OR=0.36, 95% CI=0.16, 0.82, p-value=0.015). Participants with COVID-19 illness had more than double the odds identifying as Hispanic if they were making $25,000-$75,000 (OR=2.08, 95% CI=1.20, 3.61; p-value=0.009) or $100,000-$150,000 (OR=2.41, 95% CI= 1.15, 5.04; p-value=0.019), compared to making less than $25,000 annual in household income. There were no differences in the odds of Hispanic status for those making $75,000-100,000, or $150,000-200,000, or more than $200,000, as compared to making less than $25,000 in annual household income.

Access to Care: When looking at participants with COVID-19 illness, those without health insurance had 2.67 the odds of identifying as Hispanic (OR=2.67, 95% CI=1.34, 5.29; p-value=0.005). There were no significant differences in Hispanic status and having received a COVID-19 vaccination but there were twice the odds of identifying as Hispanic for those who tested for COVID-19 (OR=2.08, 95% CI=1.33, 3.26; p-value=0.001).

Affected by COVID-19: After raking, there was no significant difference in the odds of Hispanic status for those who reported being affected by COVID-19, but participants had 2.45 the odds of identifying as Hispanic if they reported having experienced hardship due to COVID-19 (OR=2.45, 95% CI=1.51, 3.97; p-value<0.001).

Section S3: Tables of Variables, Distribution of Variables Used in Raking and Logistic Regression Models with Individual Odds Ratios Examining Adjusted Associations between Hispanic Ethnicity and Social and Environmental Factors

Table S1: Distribution of variables used in raking

**Characteristic**	Before Raking **N = 49,966**[1]	After Raking **N = 49,966**[1]
Age Group		
25-64	25,680 (51.39%)	32,977 (66.00%)
18-24	790 (1.58%)	5,996 (12.00%)
65+	23,496 (47.02%)	10,993 (22.00%)
Ethnicity		
Not Hispanic or Latino	46,517 (93.10%)	40,522 (81.10%)
Hispanic	3,449 (6.90%)	9,444 (18.90%)
Sex at Birth		
Female	32,304 (64.65%)	25,782 (51.60%)
Male	17,662 (35.35%)	24,184 (48.40%)
LGBT		
Non-LGBT	45,397 (90.86%)	44,220 (88.50%)
LGBT	4,569 (9.14%)	5,746 (11.50%)
Employment Status		
Employed	24,541 (49.12%)	30,002 (60.04%)
Retired	18,876 (37.78%)	10,426 (20.87%)
Other	6,549 (13.11%)	9,538 (19.09%)

[1]Distributions are presented as n (%). Post-raking weighted marginal distributions align with U.S. Census Bureau target estimates to the reported decimal places; a single percentage is therefore shown for each category in the After Raking column.

Table S2: COVID-19-related variables by Ethnicity

	Ethnicity			Total
**Characteristic**	**Hispanic**, N=3,449 (6.9%)[1]	**Not Hispanic**, N=46,517 (93.1%)[1]	**p-value**[2]	**N = 49,966**[1]
Received COVID-19 vaccination			<0.001	
No	2,332 (68%)	25,971 (56%)		28,303 (57%)
Yes	1,117 (32%)	20,546 (44%)		21,663 (43%)
Tested for COVID-19			<0.001	
No	1,401 (41%)	27,256 (59%)		28,657 (57%)
Yes	2,048 (59%)	19,261 (41%)		21,309 (43%)
Sick with COVID-19 symptoms			<0.001	
No	3,200 (93%)	44,866 (96%)		48,066 (96%)
Yes	249 (7.2%)	1,651 (3.5%)		1,900 (3.8%)
Affected by COVID-19			<0.001	
No	1,175 (34%)	22,454 (48%)		23,629 (47%)
Yes	2,274 (66%)	24,063 (52%)		26,337 (53%)
Experienced hardship due to COVID-19			<0.001	
No	2,678 (78%)	43,496 (94%)		46,174 (92%)
Yes	771 (22%)	3,021 (6.5%)		3,792 (7.6%)

[1]n (%)

[2]Pearson's Chi-squared test

Table S3: Ordinary and survey-weighted multivariable logistic regression models examining adjusted associations between social and environmental factors of health and Hispanic versus non-Hispanic status (Hispanic status as dependent variable).

	Before Raking				After Raking			
**Survey Question**	**OR**[12]	**SE**[2]	**95% CI**[2]	**p-value**	**OR**[12]	**SE**[2]	**95% CI**[2]	**p-value**
Age Group								
25-64	—	—	—		—	—	—	
18-24	0.96	0.111	0.77, 1.19	0.7	1.02	0.157	0.75, 1.39	>0.9
65+	0.47***	0.061	0.41, 0.52	<0.001[#]	0.43***	0.088	0.36, 0.51	<0.001[#]
Sex at Birth								
Female	—	—	—		—	—	—	
Male	0.96	0.042	0.89, 1.04	0.4	1.01	0.072	0.87, 1.16	>0.9
LGBT								
Non-LGBT	—	—	—		—	—	—	
LGBT	0.86*	0.060	0.77, 0.97	0.015	0.98	0.100	0.81, 1.19	0.9
Health Insurance								
Yes	—	—	—		—	—	—	
No	2.10***	0.085	1.77, 2.47	<0.001[#]	2.23***	0.127	1.74, 2.86	<0.001[#]
Home Ownership								
Own	—	—	—		—	—	—	
Rent	1.99***	0.045	1.82, 2.17	<0.001[#]	1.85***	0.062	1.64, 2.09	<0.001[#]

Other Arrangement	1.23**	0.079	1.05, 1.44	0.009	1.32*	0.127	1.03, 1.69	0.031
Stable House Concern								
No	—	—	—		—	—	—	
Yes	0.85**	0.060	0.76, 0.96	0.009	0.92	0.087	0.78, 1.09	0.3
Highest Education								
Never Graduated High School	—	—	—		—	—	—	
Twelve Or GED	0.20***	0.098	0.17, 0.25	<0.001#	0.25***	0.138	0.19, 0.33	<0.001#
Some College	0.14***	0.092	0.12, 0.17	<0.001#	0.19***	0.130	0.14, 0.24	<0.001#
College Graduate	0.12***	0.095	0.10, 0.14	<0.001#	0.15***	0.132	0.12, 0.20	<0.001#
Advanced Degree	0.09***	0.099	0.07, 0.11	<0.001#	0.12***	0.134	0.09, 0.15	<0.001#
Employment Status								
Employed	—	—	—		—	—	—	
Retired	0.68***	0.071	0.59, 0.78	<0.001#	0.62***	0.087	0.52, 0.73	<0.001#
Other	0.97	0.054	0.87, 1.07	0.5	0.92	0.079	0.79, 1.08	0.3
Annual Household Income								
Less than 25k	—	—	—		—	—	—	
25k - 75k	0.91	0.055	0.81, 1.01	0.077	1.09	0.084	0.92, 1.28	0.3
75k - 100k	0.82**	0.077	0.70, 0.95	0.009	1.01	0.110	0.82, 1.26	0.9
100k - 150k	0.67***	0.077	0.58, 0.78	<0.001#	0.78*	0.111	0.62, 0.96	0.022
150k - 200k	0.64***	0.100	0.52, 0.77	<0.001#	0.70**	0.132	0.54, 0.91	0.008

More than 200k	0.63***	0.095	0.53, 0.76	<0.001#	0.71*	0.142	0.54, 0.94	0.016
Received COVID vaccination								
No	—	—	—		—	—	—	
Yes	1.16***	0.042	1.07, 1.26	<0.001#	1.22***	0.059	1.09, 1.37	<0.001#
Tested for COVID								
No	—	—	—		—	—	—	
Yes	1.61***	0.038	1.49, 1.73	<0.001#	1.43***	0.055	1.28, 1.59	<0.001#
Affected by COVID								
No	—	—	—		—	—	—	
Yes	1.12*	0.048	1.02, 1.23	0.022	0.95	0.068	0.83, 1.09	0.5
Experienced hardship due to COVID								
No	—	—	—		—	—	—	
Yes	1.57***	0.055	1.41, 1.75	<0.001#	1.81***	0.079	1.55, 2.11	<0.001#
Null deviance	25,112				48,424			
Deviance	21,580				42,344			

[1]*p<0.05; **p<0.01; ***p<0.001; #p<0.01 using Holm multiplicity correction

[2]OR = Odds Ratio, SE = Standard Error, CI = Confidence Interval

Table S4: Post-raking multivariable logistic regression models examining adjusted associations between social and environmental factors of health and Hispanic versus non-Hispanic status for study participants with and without COVID-19-related illness (Hispanic status as dependent variable).

	COVID-Related Illness				No COVID-Related Illness			
**Survey Question**	**OR**[1,2]	**SE**[2]	**95% CI**[2]	**p-value**	**OR**[1,2]	**SE**[2]	**95% CI**[2]	**p-value**
Age Group								
25-64	—	—	—		—	—	—	
18-24	2.63*	0.433	1.13, 6.15	0.025	0.95	0.161	0.69, 1.30	0.7
65+	0.74	0.302	0.41, 1.33	0.3	0.42***	0.090	0.35, 0.50	<0.001[#]
Sex at Birth								
Female	—	—	—		—	—	—	
Male	0.87	0.242	0.54, 1.40	0.6	1.01	0.072	0.88, 1.17	0.9
LGBT								
Non-LGBT	—	—	—		—	—	—	
LGBT	0.73	0.348	0.37, 1.45	0.4	0.99	0.100	0.81, 1.20	>0.9
Health Insurance								
Yes	—	—	—		—	—	—	
No	2.67**	0.350	1.34, 5.29	0.005	2.25***	0.135	1.72, 2.93	<0.001[#]
Home Ownership								
Own	—	—	—		—	—	—	
Rent	1.69*	0.249	1.04, 2.76	0.034	1.87***	0.062	1.66, 2.11	<0.001[#]

Other Arrangement	1.33	0.419	0.59, 3.03	0.5	1.33*	0.131	1.03, 1.73	0.028
Stable House Concern								
No	—	—	—		—	—	—	
Yes	0.95	0.266	0.56, 1.60	0.9	0.92	0.092	0.76, 1.10	0.3
Highest Education								
Never Graduated High School	—	—	—		—	—	—	
Twelve Or GED	0.17***	0.395	0.08, 0.37	<0.001[#]	0.25***	0.144	0.19, 0.33	<0.001[#]
Some College	0.08***	0.359	0.04, 0.16	<0.001[#]	0.20***	0.137	0.15, 0.26	<0.001[#]
College Graduate	0.08***	0.387	0.04, 0.18	<0.001[#]	0.16***	0.139	0.12, 0.21	<0.001[#]
Advanced Degree	0.04***	0.430	0.02, 0.09	<0.001[#]	0.12***	0.141	0.09, 0.16	<0.001[#]
Employment Status								
Employed	—	—	—		—	—	—	
Retired	0.36*	0.414	0.16, 0.82	0.015	0.63***	0.090	0.53, 0.75	<0.001[#]
Other	1.20	0.258	0.73, 2.00	0.5	0.90	0.082	0.77, 1.06	0.2
Annual Household Income								
Less than 25k	—	—	—		—	—	—	
25k - 75k	2.08**	0.280	1.20, 3.61	0.009	1.03	0.087	0.87, 1.22	0.7
75k - 100k	1.73	0.440	0.73, 4.09	0.2	0.97	0.113	0.78, 1.21	0.8
100k - 150k	2.41*	0.376	1.15, 5.04	0.019	0.72**	0.115	0.57, 0.90	0.004
150k - 200k	2.30	0.663	0.63, 8.45	0.2	0.65***	0.129	0.50, 0.83	<0.001[#]

More than 200k	1.23	0.613	0.37, 4.10	0.7	0.67**	0.139	0.51, 0.87	0.003
Received COVID vaccination								
No	—	—	—		—	—	—	
Yes	1.09	0.235	0.69, 1.73	0.7	1.25***	0.061	1.11, 1.41	<0.001[#]
Tested for COVID								
No	—	—	—		—	—	—	
Yes	2.08**	0.227	1.33, 3.26	0.001[#]	1.38***	0.056	1.24, 1.54	<0.001
Affected by COVID								
No	—	—	—		—	—	—	
Yes	0.96	0.257	0.58, 1.59	0.9	0.95	0.069	0.83, 1.08	0.4
Experienced hardship due to COVID								
No	—	—	—		—	—	—	
Yes	2.45***	0.247	1.51, 3.97	<0.001[#]	1.75***	0.082	1.49, 2.06	<0.001[#]
Null deviance	2,866				45,355			
Deviance	2,295				39,873			

[1]*p<0.05; **p<0.01; ***p<0.001; [#]p<0.01 using Holm multiplicity correction

[2]OR = Odds Ratio, SE = Standard Error, CI = Confidence Interval

Table S5: Description of variables used in the study.

Variables are grouped into their respective sources from the *All of Us* Research Data. The third column provides the values or levels of each variable allowed on the survey, while the last column provides the final values/levels used after applying some form of variable encoding or transformation.

**Variable Name**	**Full description or survey question**	**Possible values or levels**	**Binning/Levels Used**
	**The Basics Survey**		
Sex at birth	What was your biological sex assigned at birth?	Female, Male, Intersex, None, Prefer not to answer, No matching concept, Skip	Female, Male
Gender identity	What terms best express how you describe your gender identity? (Check all that apply)	Woman, Man, Non-Binary, Transgender, Additional Options, Prefer not to answer	Woman, Man, Other (multiple, non-binary, transgender, none of these)
Sexual orientation	Which of the following best represents how you think of yourself?	Straight, Bisexual, Gay, Lesbian, None, Prefer not to answer, Skip	Straight, Sexual Minority (Bisexual, Gay/Lesbian, Multiple, None)
LGBT	A derived variable, defined to include sexual and gender minority groups.	LGBT, non-LGBT	LGBT, non-LGBT
Date of birth	Date of birth	Date string	
Age/Age groups	Derived from date of birth and the date the COPE survey (i.e., survey_datetime variable) was	Integer values ranging from 18 to 104 (in years)	18-24,<br>25-64,<br>65+

	completed by each participant.		
Race	Race	White, Black or African American, Middle Eastern or North African, Native Hawaiian or other Pacific Islander, More than one population, None Indicated	**White, Black or African American, Asian** (Asian, Native Hawaiian or Other Pacific Islander), **Other** (Middle Eastern or North African, More than one population, None Indicated)
Ethnicity (the dependent variable)	Ethnicity	Hispanic or Latino, Not Hispanic or Latino, No matching concept, No of these, Prefer not to answer, Skip	Limited sample size to **Hispanic** or Latino and **Not Hispanic**
Home ownership	Do you own or rent the place where you live?	Own, Rent, Other arrangement with branching logic (not considered)	Own, Rent, Other Arrangement
Health Insurance	Are you covered by health insurance or some other kind of healthcare plan?	Yes with a branching logic (not considered), No, Don't know, Prefer not to answer	Yes/No
Stable house concern	In the past 6 months, have you been worried or concerned about NOT having a place to live?	Yes/No	Yes/No
Annual income	What is your annual household income from all sources? This includes personal income plus the income of all family members in the household for the last	Less than $10,000<br>$10,000- $24,999<br>$25,000-$34,999<br>$35,000-$49,999<br>$50,000- $74,999<br>$75,000-$99,999<br>$100,000-$149,999	**Less than 25k** (less 10k, 10k-25k), **25k-75k** (25k-35k,35k-50k,50k-75k),<br>**75k - 100k,**<br>**100k - 150k,**<br>**150k - 200k,**

	calendar year. Also includes all wages and other sources of income.	$150,000-$199,999<br>$200,000 or more	**More than 200k**
Highest education	What is the highest grade or year of school you completed?	● Never attended school or only attended kindergarten<br>● Grades 1 through 4 (Primary)<br>● Grades 5 through 8 (Middle school)<br>● Grades 9 through 11 (Some high school)<br>● Grade 12 or GED (High school graduate)<br>● 1 to 3 years after high school (Some college, Associate's degree, or technical school)<br>● College 4 years or more (College graduate)<br>● Advanced degree (Master's, Doctorate, etc.)<br>● Prefer not to answer	**Never Graduated High School** (Never Attended, One Through Four, Five Through Eight, Nine Through Eleven),<br>**Some College** (College One to Three),<br>**Twelve or GED, College Graduate, Advanced Degree**
Employment status	What is your current employment status? Multiple responses are allowed.	● Employed for wages (part- time or full-time),<br>● Self-employed,<br>● Out of work for 1 year or more,<br>● Out of work for less than 1 year,<br>● A homemaker,<br>● A student,<br>● Retired,	**Employed** (Self-employed, employed for wages),<br>**Retired**,<br>**Other** (Unable to work, homemaker, student, Out of work less than 1 year, out of work more than 1 year), along with any

		● Unable to work (disabled),<br>● Prefer not to answer	other combination of choices
**COPE Survey**			
Tested for COVID-19	Were you tested for COVID-19 in the past month?	Yes with sub-questions but ignored, No, No I tried and was unable to be tested, I don't know	Yes/No
Vaccinated	Did you receive the COVID-19 vaccination?	Yes with sub-questions but ignored, No, Not sure, I participated in a COVID-19 vaccination trial	Yes/No
Sick with COVID-19	In the past month, have you been sick for more than one day with a new illness related to COVID-19 or flu-like symptoms?	Yes, No, Skip	Yes/No
Affected by COVID-19<br><br>(Converted to a binary variable of not affected or affected by at least one of the conditions listed)	In the past month, how has the COVID-19 outbreak affected you? Please select all that apply.	● Worked remotely or from home more than you used to before COVID-19<br>● Worked more hours than usual<br>● Worked reduced hours<br>● Was not able to work due to COVID-19 related illness<br>● I became unemployed<br>● Had difficulty arranging for childcare	Yes (at least one of the given options),<br><br>No (None of the given options)

		● Incurred increased costs for childcare expenses<br>● Worked with children at home with me<br>● Income or pay has been reduced<br>● Not paid at all<br>● Had serious financial problems<br>● None of the above	
Experienced hardship<br><br>(Converted to a binary variable of no hardship or at least one hardship)	In the past month, have you experienced the following as a result of COVID-19? Select all that apply.	Not enough money to pay rent<br>● Not enough money to pay for gas<br>● Not enough money to pay for food<br>● Not enough money to pay for medications<br>● Did not have a regular place to sleep or stay<br>● None of the above	Yes (at least one of the given options),<br><br>No (None of the given options)
